# Investigating associations between allostatic load phenotypes and clinical impairment in youth with chronic pain

**DOI:** 10.1016/j.bbih.2026.101297

**Published:** 2026-07-06

**Authors:** Aahir Mrittika, Catherine Ireland, Carter R. Petty, Michelle Bosquet Enlow, Sarah Nelson

**Affiliations:** a Department of Anesthesiology, Critical Care, and Pain Medicine, Boston Children's Hospital, USA; b Boston University Aram V. Chobanian & Edward Avedisian School of Medicine, USA; c Biostatistics and Research Design Center, Boston Children's Hospital, USA; d Department of Psychiatry and Behavioral Sciences, Boston Children's Hospital, USA; e Department of Psychiatry, Harvard Medical School, USA

**Keywords:** Allostatic load, Chronic pain, Clinical phenotypes, Pediatric health

## Abstract

Allostatic load (AL), defined as nervous system wear and tear in response to repeated or prolonged stress, has been hypothesized to underlie risk for the onset and/or maintenance of chronic pain. However, minimal research has directly examined the measurement and interpretation of AL in relation to chronic pain in clinical populations. Recent work in a community sample of adults suggests relations between chronic pain and “allostatic load phenotypes” (e.g. parasympathetic dysregulation and metabolic dysregulation), where the metabolic dysregulation phenotype showed to predict greater pain interference and a higher number of pain sites compared to low allostatic load phenotype. Given the dearth of understanding on how AL manifests in youth with chronic pain, the current study aimed to investigate AL phenotypes in youth with chronic pain and their associations with clinical outcomes. Allostatic load measures, including salivary cortisol, dehydroepiandrosterone (DHEA), and C-reactive protein, as well as waist–hip ratio, body-mass index, and blood pressure, were collected during previously scheduled new patient evaluations at a tertiary pain clinic. Results indicate biomarkers related to cardiovascular and cortisol phenotypes show good fit with the data. Further, youth with high cardiovascular risk and low cortisol risk evidenced greater pain catastrophizing, and those with high Cortisol risk evidenced greater exposure to childhood adversity. Future research should continue to examine the manifestation of these phenotypes in larger and broader chronic pain populations in youth and capture how these phenotypes may respond to intervention.

## Highlights

• Allostatic load – nervous system “wear and tear” in response to stress – may underlie chronic pain morbidity, but this remains understudied.
• The current investigation applies a model of allostatic load phenotypes developed in an adult sample to youth with chronic pain.
• Results indicate cardiovascular and cortisol phenotypes provide best fit in a pediatric chronic pain sample.
• Future research should examine allostatic load biomarkers and phenotypes in larger, more diverse chronic pain samples.

## 1 Introduction

Chronic pain, or pain lasting three months or longer in the absence of injury or tissue damage (Fillingim et al., 2014), has a prevalence of 20.8% in pediatric populations (Chambers et al., 2024), and is associated with notable morbidity and healthcare burden (van Hecke et al., 2013; Korterink et al., 2015; Manners, 2017). Due to the well documented association between childhood adversity and chronic pain (Devanarayana et al., 2012; Heim et al., 1998; Jones et al., 2009; Nicol et al., 2016; Huffhines and Jackson, 2019; Schofferman et al., 1993; Sansone et al., 2013; Nelson et al., 2017, 2021a), it has been proposed that a maladaptive physiological stress response (e.g., glucocorticoid, cardiovascular dysregulation (Lupien et al., 2015)) may underlie the onset (Abdallah and Geha, 2017; Nelson et al., 2020a) and chronicity of pain (Nelson et al., 2016, 2020a, 2021b, 2022). Moreover, given the heightened plasticity of neurobiological stress systems in childhood and adolescence (Palermo et al., 2014), addressing stress physiology in youth with chronic pain via mechanistically informed treatments may enhance treatment outcomes into adulthood (Nelson et al., 2021c, 2024). However, the measurement and characterization of the physiological stress response (e.g., cortisol, heart rate variability (Whelan et al., 2021)) in youth with chronic pain is significantly understudied and poorly characterized, hampering efforts to develop relevant risk-identification profiles and targeted interventions.

Allostatic load (AL), defined as nervous system wear and tear in response to repeated or prolonged stress (Lupien et al., 2009, 2015; Juster et al., 2010; McEwen, 1998), has been hypothesized to underlie risk for the onset and/or maintenance of chronic pain conditions (e.g., migraine, fibromyalgia) (Nelson et al., 2016, 2020a; Borsook et al., 2012). Notably, evidence of AL, including dysregulation of the Hypothalamic-Pituitary-Adrenal (HPA) axis (Heim et al., 1998; Bomholt et al., 2004), and glucocorticoid (Hannibal and Bishop, 2014; Woda et al., 2016), cardiovascular (Evans et al., 2013), and lipid dysfunction (Santos et al., 2017), has been found in chronic pain patients, but minimal research has explored the full construct of AL, which is captured best when modeling multisystem physiological dysregulation (Wiley et al., 2016). A pilot investigation examining associations between AL risk factors and pain-related outcomes in youth with chronic pain showed that greater than 50% of the sample evidenced “high risk” (2 or more risk indicators) for AL; further, findings suggested that AL risk may moderate associations between psychological and physical impairment in these youth (Nelson et al., 2021b). However, beyond this initial study, minimal research has explored the measurement and interpretation of AL in relation to chronic pain in youth.

Recent work by Liang et al., in a large community sample of adults identified “allostatic load phenotypes” that showed differential associations with chronic pain risk, pain distribution, and pain interference (Liang and Booker, 2024). A latent class analysis (a) revealed three distinct biomarker clusters in the sample – low biological dysregulation, parasympathetic dysregulation, and metabolic dysregulation – with the “metabolic dysregulation” phenotype predicting greater pain interference and a higher number of pain sites compared to the phenotype of low biological dysregulation. The low biological dysregulation phenotype demonstrated low risk across most biomarkers, functioning as a 'baseline' phenotype for comparison. These findings suggest it may be useful to similarly identify and examine allostatic load phenotypes in youth with chronic pain, especially given the breadth of clinical phenotypes in pediatric pain care today (Chambers et al., 2024). There is an ongoing need for more meaningful ways to characterize and enhance early diagnosis, prevention, treatment, and prognosis in these populations (Chambers et al., 2024; Carter and Threlkeld, 2012; Cunningham and Kashikar-Zuck, 2013; Cunningham et al., 2019; Nelson et al., 2020b), as they are in a critical stage of development (Frodl and O'Keane, 2013). Diagnosing or detecting risk factors early offer hope for intervention optimization long-term (Palermo et al., 2014; Noel et al., 2015). The overall goal of the current study was to (1) identify AL phenotypes in youth with chronic pain (Liang and Booker, 2024) and (2) examine their associations with clinical outcomes. We anticipate that the findings from this study can be applied to enhance the growing body of research on stress-based physiological dysregulation in relation to pain chronicity and long-term outcomes in youth. The ultimate goal of this line of work is to guide future research in prevention and mechanistically informed treatment development for these debilitating conditions.

## 2 Methods

### 2.1 Participants

The current investigation (IRB-P00027928) analyzed data collected for a previously published study that assessed the feasibility of a multifactorial AL model in predicting pain and related impairments (Nelson et al., 2021b). Participants were aged 10-17 and presented for an initial evaluation at the pain treatment service (PTS) clinic at Boston Children's Hospital. Participants with a specific disease-based diagnosis e.g. sickle cell disease, severe cognitive impairment, and insufficient fluency in English to participate in the surveys were excluded. Potential participants were contacted prior to their scheduled appointment and offered the option to participate in the study. If they indicated interest, they were contacted again 48 h prior to their appointment, at which time formal consenting and assenting procedures were conducted with the participant and in the presence of a legal parent/guardian followed by the review of questionnaire and saliva collection procedures. Home collection of three saliva samples was requested to take place in the morning of their appointment immediately upon awakening (two samples) before eating, drinking, or brushing teeth, and then again 30 min later (one sample). Upon presentation to their clinical appointment, participants were asked to provide their home-collected saliva samples and saliva collection diary for labeling and storage, asked to complete self-report questionnaires via REDCap (Research Electronic Data Capture (Harris et al., 2019)), and then escorted to a confidential exam room where their physiological measures were recorded (i.e., blood pressure, waist-hip ratio [WHR], body-mass index [BMI]) by a Registered Nurse (RN) or trained study staff member. Participants were provided with a $10 gift card for study participation.

### 2.2 Outcome measures

#### 2.2.1 Demographic information

Parents were asked to report participants’ age, sex, and race/ethnicity. Participants and their parentss also reported on the frequency and time since the first onset of pain symptoms, and the nature or location of their pain complaint.

#### 2.2.2 Allostatic load biomarkers

HPA Axis Functioning: Cortisol and dehydroepiandrosterone (DHEA) were assessed from three saliva samples collected via passive drool at home prior to the participant's scheduled clinical appointment. Cortisol 1 and DHEA were assayed from the two saliva samples collected via passive drool into SaliCap tubes immediately upon waking prior to eating, drinking, or brushing teeth; cortisol 2 was collected 30 min later (also before eating, drinking, or brushing teeth). All measures were log-transformed before the analysis.

Cardiovascular Functioning: Systolic and diastolic blood pressures (SBP, DBP) and resting heart rate (HR) were collected by a Registered Nurse (RN) at the clinic appointment as part of the standard vitals.

Metabolic Dysregulation: Physical measures were collected by a trained research coordinator in a private exam room during the patient's appointment. BMI and waist-to-hip ratio were calculated from the collected measures.

Inflammation: C-reactive protein (CRP), a broad metric that has been used to represent systemic inflammation (Pay and Shaw, 2019), was measured via assaying the first saliva sample collected immediately after waking up. In order to minimize participant burden and given the lack of extant data on inflammation in youth seeking specialty pain care, CRP as a broad metric was prioritized over the spectrum of inflammatory cytokines.

#### 2.2.3 Pain-related and psychosocial functioning

*Numeric Rating Scale (NRS) Pain Intensity—Child* Report (von Baeyer et al., 2009) is a pain intensity score (0–10 scale) over the last two weeks where “0” indicates no pain and “10” indicates the worst possible pain. It is categorized into “usual” (i.e., average or typical), “best” (i.e., lowest), and “worst” (i.e., highest). The “worst” and “usual” pain scores were highly correlated (r = 0.752, p < .001), and so a factor analysis was run to avoid redundancy and create a single construct reflecting pain burden. “Best” pain was excluded due to a substantially lower factor loading (0.431) relative to other two items in the initial factor analysis. The remaining two scores were re-entered into the factor analysis, both loading onto a common factor (loadings = 0.867), and their factor scores were used to create a standardized pain intensity score.

*Functional Disability Inventory (FDI* (Claar and Walker, 2006; Walker and Greene, 1991)*)* is a self-report measure of perceived difficulty in performing activities in school, home, physical, and social contexts. It has been validated for children ages 8–17. Cutoff scores are as follows: 0–12—no/minimal disability; 13–29—moderate disability; 30+—severe disability. Participants completed this measure as part of their regularly scheduled clinical appointment.

*PROMIS Pediatric Short Forms* (Jacobson et al., 2013) are validated self-report questionnaires that ask about a variety of aspects related to child/adolescent physical, mental, and social health. Raw score totals on each measure can be converted to T-scores, with higher scores indicative of greater impairment. For the current study, the Sleep Disturbance (8 items), Anxiety (8 items), Depressive Symptoms (8 items), and Psychological Stress (8 items) pediatric self-report short forms (ages 8–17) were administered during the clinical appointment.

*Childhood Trust Events Survey (CTES* (Pearl, 2000)*)* is a questionnaire designed to screen for a variety of lifetime stressful life events, including adverse childhood experiences (ACEs), in youth. Items are scored as yes/no for the experience of an event and summed for a total exposure score, with higher scores indicating greater exposure to stressful life events. For the purposes of the current study, only the parent-report version was administered, but youth and caregivers were asked to complete it collaboratively to obtain a more comprehensive assessment of participant adversity exposures (Felitti et al., 1998).

### 2.3 Data analysis

All analyses were performed using R.4.1 (Team, 2021). Partial correlations, controlling for age and sex, tested associations between individual allostatic load biomarkers and primary outcomes, to determine if the variables were sufficiently correlated (*r* > .30 Tabachnick and Fidell, 2013) prior to a factor analysis.

The present study adapted the methodology of Liang et al. (2024) (Liang and Booker, 2024) to a pediatric sample, retaining their risk-classification approach and factor analysis framework. The original study organized 27 AL biomarkers into 7 physiological systems (e.g. cardiovascular, immune, metabolic) and dichotomized the biomarkers using the sample 25th percentile as the cutoff. “High risk” or “1” was assigned to values in the upper or lower 25th quartile depending of the direction of risk for biomarker, and “0” otherwise. Latent Class Analysis (LCA) applied to these binary biomarkers yielded three distinct phenotypes in the original adult sample: metabolic dysregulation, low biological dysregulation, and parasympathetic dysregulation.

In the present study, the same risk-classification framework was applied to the pediatric sample; however, rather than replicating the LCA used in the adult sample, a Confirmatory Factor Analysis (CFA) was selected to test whether the hypothesized factor structure would be supported in this population. Reliable analysis using the LCA would require a substantially larger sample than the present study (Wurpts and Geiser, 2014-). Analyses were conducted in R using the lavaan package (Rosseel, 2012, 2018). The biomarkers were defined for each factor based on existing literature (Juster et al., 2010; Liang and Booker, 2024; Carbone et al., 2022): HPA axis functioning included Cortisol 1, Cortisol 2, and DHEA; cardiovascular functioning included systolic blood pressure (SBP), diastolic blood pressure (DBP), and resting heart rate (HR); and metabolic dysregulation included BMI and WHR. As CRP was the only inflammation-related biomarker, with other biomarkers such as Interleukin-6 (IL6) from the original study missing from our sample, an inflammation factor could not be constructed. CRP was therefore retained as an individual biomarker in the subsequent analyses. While choosing the biomarkers for each construct or factor was based in AL theory, biomarkers that loaded poorly on the factors (r < 0.50) were excluded from the combined constructs or factors to ensure construct validity (Hair et al., 2010). They were instead treated as individual risk factors.

In replication of Liang et al. (Liang and Booker, 2024), participants with biomarker levels in the upper 25th quartile were classified as high risk and coded as 1, and participants in the other three quartiles were classified as low risk and coded as 0. Exceptions to this coding scheme were for DHEA and cortisol measures, where participants who fell in the *upper or lower* 25th quartiles were classified as high risk to detect a dysregulated HPA axis. Participants at high-risk for the any of the biomarkers loading strongly on factors in the CFA were assigned high-risk status for the corresponding factor. All risk-dichotomized biomarkers were then fit as predictors in simple linear regression models with pain and psychosocial outcomes as the dependent variables.

To examine whether biological sex influenced the results, all regression analyses and the CFA were repeated in biological females only. Model fit indices showed negligible differences from the full-sample solution, and statistically significant relationships remained consistent across both sets of analyses. Sex was therefore not included as a covariate in the final models.

## 3 Results

Data from 61 participants (*M*age = 14.51, sd = 1.9) were used. Participants were predominantly female identifying (88.5%) and white/Caucasian (88.5%). Table 1 details participant sociodemographic and clinical characteristics. Individual quartile thresholds for each biomarker are detailed in Table 2. Participants most commonly reported widespread or multiple pain locations, followed by complex regional pain syndrome (CRPS) or amplified musculoskeletal pain syndrome (AMPS). CTES asks for physical abuse, homelessness, neglect, etc. , and in our sample,bullying had the highest prevalence (38.6%), followed by mental illness of a family member (31.7%)., and painful/frightening medical treatment (29.5%).

**Table 1:** Sample characteristics; *N* = 61.

Age	14.47 (1.96)	10 – 17
Sex (*n*, %)		
Female	54 (88.5)	
Male	6 (9.8)	
Other	1 (1.6)	
Race/Ethnicity (*n*, %)		
White	54 (88.5)	
Black	3 (4.9)	
Asian	1 (1.6)	
Hispanic or Latino	1 (1.6)	
Other	2 (3.3)	
PCS-C	25.74 (12.61)	4 – 47
Pain intensity		
Usual Pain	6.43 (1.87)	0 – 10
Worst Pain	8.04 (1.47)	4 – 10
FDI	21.82 (11.78)	0 - 50
PROMIS Measures		
PROMIS Anxiety	50.76 (10.67)	33.5 – 74.6
PROMIS Depressive Symptoms	52.72 (9.94)	35.2 – 71.4
PROMIS Psychological Stress	56.96 (9.28)	37 – 81.8
PROMIS Sleep Disturbance	60.14 (7.46)	46.8 – 78.5
CTES – Adversity Exposure	2.10 (2.01)	0 - 7

*Abbreviations:* CTES, Childhood Trust Events Survey; FDI, Functional Disability Inventory; PCS-C, Pain Catastrophizing Scale (child version). Variables are presented as mean (standard deviation) with their respective ranges, or n (%).

**Table 2:** High risk quartiles.

Phenotype	Quartile Cut-offs for High Risk
Cardiovascular	
Systolic	≥ 124 mm Hg
Diastolic	≥ 77.0 mm Hg
Heart Rate	≥ 94.0 bpm

Cortisol (log-transformed)	
Cortisol 1∗	≤ 1.45 or ≥ 2.54
Cortisol 2∗	≤ 2.19 or ≥ 2.77

Individual Biomarkers (log-transformed)	
BMI	≥ 25.3
Waist-Hip ratio	≥ 0.802
CRP	≥ 2.62
DHEA	≤ 4.87 or ≥ 6.15

*Abbreviations:* BMI, Body Mass Index; CRP, c-reactive protein, DHEA, dehydroepiandrosterone; BPM, beats per minute; mm Hg, millimeters of mercury.

Note. ∗*Cortisol 1* is the measurement of cortisol upon awakening *Cortisol 2* is the measurement of cortisol 30 min after *Cortisol 1*.

Lower (25th percentile) and upper quartiles (75th percentile) are included in the high-risk thresholds for Cortisol and DHEA.

Partial correlation analyses, controlling for age and sex, revealed several significant associations among the continuous AL biomarkers and clinical outcomes. Specifically, biomarkers within category showed moderate to strong correlations with each other: HPA axis functioning: cortisol 1 and cortisol 2 (*r* = 0.72, *p* < .001); cardiovascular functioning: systolic heart rate and diastolic heart rate (*r* = 0.71, *p* < .001); metabolic dysfunction: BMI and WHR (*r* = 0.50, *p* = .02). All partial correlations are shown in Table 3.

**Table 3:** Partial correlations controlling for age and sex.

Variable	1	2	3	4	5	6	7	8	9	10	11	12	13	14	15	16	17
1. Heart Rate	--																
2. Systolic	0.54∗∗	--															
3. Diastolic	0.58∗∗	0.71∗∗∗	--														
4. BMI	0.18	0.57∗∗	0.31	--													
5. WHR	0.18	0.11	0.14	0.50∗	--												
6. CRP	0.16	0.39	0.19	0.53∗	0.36	--											
7. Cortisol 1	0.13	0.25	0.27	0.06	0	0.18	--										
8. Cortisol 2	0.24	0.32	0.34	−0.04	−0.1	0.34	0.72∗∗∗	--									
9. DHEA	0.32	0.61∗∗	0.54∗∗	0.37	0.09	0.25	0.46∗	0.5∗	--								
10. FDI	0.39	0.23	0.4	−0.26	−0.15	−0.2	0.22	0.27	0.09	--							
11. PROMIS sleep	0.25	0.02	0.13	0	−0.12	−0.34	0.23	0.08	0.29	0.48∗	--						
12. PROMIS Stress	0.13	−0.04	0.01	−0.36	−0.33	−0.28	0.2	0.04	0.02	0.44∗	0.55∗∗	--					
13. PROMIS Depressive Symptoms	0.39	0.07	0.1	−0.36	−0.28	−0.17	−0.07	−0.04	−0.01	0.42∗	0.5∗	0.84∗∗∗	--				
14. PROMIS Anxiety	0.16	−0.1	−0.18	−0.18	−0.17	−0.16	0.17	0.06	0.06	0.42	0.69∗∗∗	0.8∗∗∗	0.71∗∗∗	--			
15. Pain Intensity	−0.05	0.1	0.29	0.02	0.26	−0.15	0.28	0	0.17	0.48∗	0.27	0.29	0.03	0.28	--		
16. Childhood Adversity	−0.04	−0.05	−0.06	0.05	−0.3	−0.15	0.17	−0.03	0.14	0.32	0.48∗	0.41	0.19	0.54∗∗	0.3	--	
17. PCS	0.29	0.38	0.47∗	0.01	0.01	0.16	0.24	0.27	0.09	0.44∗	0	0.36	0.23	0.16	0.53∗	0.06	--

Note: ∗p < .05, ∗∗p < .01, and ∗∗∗p < .001. *Abbreviations:* BMI, Body Mass Index; DHEA, Dehydroepiandrosterone; FDI, Functional Disability Inventory; *PCS*-C, Pain Catastrophizing Scale (child version); PROMIS, Patient-Reported Outcomes Measurement Information System; WHR, Waist-hip Ratio.

A two-factor model CFA model demonstrated excellent fit to the data, χ^2^ (5) = 2.18, *p* = .824, and fit indices indicated strong model adequacy (CFI = 1.000, TLI = 1.086, RMSEA = 0.000, 90% CI [0.000, 0.110], SRMR = 0.039). The cardiovascular phenotype included systolic blood pressure, diastolic blood pressure, and resting heart rate. Due to its weak loading on the common HPA axis factor relative to the cortisol measures (standardized loading = 0.335), log-transformed DHEA was modeled as an independent risk factor to preserve the integrity of the factor structure. The HPA axis phenotype comprised Cortisol 1 and Cortisol 2 only and was renamed the cortisol phenotype. BMI and WHR did not load strongly on a common metabolic phenotype; thus, they were treated as separate risk factors. CRP was treated as an individual risk factor as well, with no other indicators for an inflammation phenotype.

**Fig. 1:**
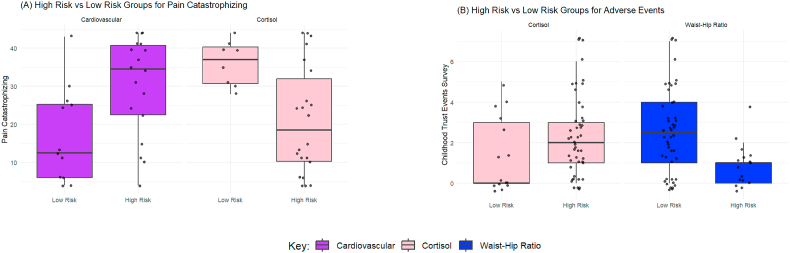
**Statistically Significant Risk Groups.** (A) Cardiovascular (*p* = .010) and Cortisol (*p* = .007) phenotypes for Pain Catastrophizing. (B) Cortisol (*p* = .042) and Waist-Hip Ratio (*p* = .009) for Childhood Adversity.

Biomarkers for the cortisol and cardiovascular phenotypes loaded significantly onto their respective latent factors, with standardized loadings ranging from 0.820 to 0.824 for the cortisol factor and 0.563 to 0.833 for the cardiovascular factor. The covariance between the cortisol and cardiovascular factors was not significant (r = 0.045, *p* = .789). When examining relations among phenotypes and individual risk factors with clinical outcomes, results suggested that participants who were in the high risk group for the cardiovascular phenotype showed higher levels of pain catastrophizing, *b* = 14.53, *t*(28) = 3.24, *p* = .003. Participants in the high risk cortisol phenotype reported higher childhood adversity *b* = 1.24, *t*(59) = 2.08, *p* = .042, and lower pain catastrophizing scores, *b* = −15.09, *t*(28) = −2.89, *p* = .007, compared to those in the low risk group. Participants in the high risk WHR group reported lower rates of adversity exposure, *b* = −1.54, *t*(59) = −2.71, *p* = .009. Fig. 1 shows the different risk biomarkers graphed against significant outcomes. BMI, CRP, and DHEA did not show significant associations with any of the outcomes.

## 4 Discussion

Emerging literature suggests that youth with chronic pain report higher levels of stress and adversity exposure and poorer psychosocial functioning compared to their non-pain peers (Nelson et al., 2018, 2020b; Kashikar-Zuck et al., 2010). Initial studies have begun to elucidate potential shared mechanisms underlying pain and psychosocial stress, including biomarkers of allostatic load (AL) – nervous system “wear and tear” evidenced across multiple stress regulatory systems. In this area, Nelson and colleagues (2021) found that greater than 50% of youth with chronic pain evidenced high AL risk (Nelson et al., 2021b), compared to a 19.7% high AL prevalence found in a nationally representative sample of adolescents (Mathis et al., 2025). Recent research in adults has identified distinct phenotypes of AL – namely, low biological dysregulation, parasympathetic dysregulation, and metabolic dysregulation. These outcomes provide new meaning to the construct with real, actionable avenues to support catered clinical care – i.e., adults that evidenced the cardiovascular phenotype were at higher risk for poorer function (Liang and Booker, 2024), which underscored the necessity of focusing on cardiovascular function in treatment.

Broadly examining the common variability of AL biomarkers (e.g., phenotypes) can help understand mechanisms in which biomarkers respond together within biological systems to shape clinical outcomes (Liang and Booker, 2024), which can help in greater understanding of the consequences of prolonged stress. In contrast to a composite system that integrates multiple AL biomarkers, studying distinct systems may enable more targeted recognition and intervention pathways. Additionally, this approach may help preserve system-level information that could otherwise be lost in a composite measure.

The present study adopted the framework of a factor analysis by Liang et al. (2024) (Liang and Booker, 2024), to replicate the outcomes in a pediatric chronic pain sample. In our sample of treatment-seeking youth with chronic pain, the two factors (i.e., “phenotypes”) that emerged were a cortisol factor and a cardiovascular factor*,* with additional individual biomarkers of BMI, WHR, and DHEA that did not load onto any common factor. Given that BMI and WHR were moderately correlated, as were DHEA and the cortisol measures, the biomarkers in each group may share some common variance with each other but may not be fit for a factor analysis in our cohort. This could indicate either the appropriateness of coding AL as one construct using multiple biomarkers (which is historically what has been done (Wiley et al., 2016; Rogosch et al., 2011)) or suggest that a larger sample size may be needed to capture the underlying constructs they are hypothesized to share. Further research should address this question.

Following the coding scheme outlined in the model paper with adults, we categorized “high risk” for cortisol using the *lowest and highest* quartiles across the sample. This approach accounts for the bidirectional nature of HPA dysregulation, as both elevated and blunted cortisol levels are associated with adverse physiological outcomes (George et al., 2025). Here we found that participants in the high-risk cortisol phenotype reported relatively lower levels of pain catastrophizing. Although these findings diverge from the typical “high stress – high impairment” presentation documented in pediatric pain populations (Nelson et al., 2020b), one potential explanation may be HPA-axis dysregulation in the *absence of self-reported* catastrophizing. Defensiveness or repression are broad psychological constructs that may lead to underreporting of psychological symptoms, common in treatment-seeking youth with chronic pain (Franklin et al., 2016; Ruskin et al., 2023) (one study found over 30% in the sample). This may be further compounded by ‘social desirability’ (Logan et al., 2008) bias, meaning that their symptom repression is driven by the desire to present more socially positive characteristics (Ruskin et al., 2023). Children could also be underreporting distress so their pain is taken more seriously (Logan et al., 2008), as many families feel pushed towards mental health treatment when they are seeking more medicalized interventions (Defenderfer et al., 2018; Neville et al., 2019). It should be noted that the present study did not assess repression or social desirability, and these are offered as speculative explanations for the observed discrepancy between limited self-reported distress and evidence of dysregulated cortisol. These findings raise the possibility that lower catastrophizing scores do not always reflect better coping, and future research should examine the broader implications of HPA-axis dysregulation in the absence of self-reported distress.

The high-risk cortisol group also reported greater childhood adversity exposure, consistent with evidence that repeated early stressors can disrupt HPA axis functioning and result in either overactivated or suppressed cortisol patterns (Carrion et al., 2002; Pan et al., 2018; Danese and McEwen, 2012; Counts et al., 2022; Brindle et al., 2022). The CTES checks for ACEs including physical abuse, homelessness, and neglect, with bullying as the most frequently reported adversity in our sample. Given the well-established association between ACEs and chronic pain (Nelson et al., 2018, 2021a), accurate cortisol measurement in pediatric populations is especially important for guiding targeted intervention. Psychological resilience has emerged as an area of interest, as a potential mechanism to mitigate symptoms of HPA dysregulation in adversity-exposed youth (Chaimano et al., 2025; Nishimi et al., 2022). Relatedly, specific ACEs have been differentially associated with elevated and blunted cortisol ‘reactivity’ (measured after a specific task), raising important questions regarding the heterogeneity of biomarker profiles and stress responses in this population (Iob et al., 2021).

Conversely, higher pain catastrophizing was observed in the high-risk cardiovascular phenotype group. Pain catastrophizing has been previously associated with elevated SBP and DBP in adult chronic pain patients (Leonard et al., 2013), and our results may be capturing a similar biological symptom of heightened pain-related anxiety and subsequent nervous system reactivity in youth. The results highlight the role of a psychosocial factor such as catastrophizing being direct a risk factor in cardiovascular disease and hypertension risk outcomes, and it may be meaningful to investigate how alleviating catastrophizing symptoms may impact cardiovascular health.

Among individual biomarkers, participants in the low risk WHR category reported higher ACEs, which have been previously linked to both obesity and underweight (Hanć et al., 2022), as well as eating disorder symptoms and dysregulated eating behaviors (Hanć et al., 2022; Guillaume et al., 2016; Rienecke et al., 2022). Given that being underweight is also associated with elevated mortality (Roh et al., 2014) and depression (De Wit et al., 2009), future research should examine metabolic AL indicators from a bidirectional risk perspective in this population.

Strengths of the current study include its consideration of AL in a pediatric chronic pain population, an understudied but promising area. Youth with chronic pain are susceptible to prolonged stress and AL; thus, applying a systemic approach to how AL manifests physically may be beneficial in clinical identification and intervention early in life. We focused on implementation of minimally burdensome biospecimen collection methods to maximize acceptability and compliance in this vulnerable, severely understudied patient population. Statistically validated questionnaires collected in a clinical context likely optimized the validity of our health outcomes. The major limitation of the current study is the small sample size, which limited efforts to conduct exploratory factor analyses that can uncover latent constructs. Limited data on participant medication usage may have impacted the accuracy of our interpretations. In the measures, the Childhood Trust Events Survey (CTES) was filled out collaboratively between the parent and the child and may not include events the youth does want to report in the presence of a parent. Furthermore, the sample was predominantly female and White, which limits generalizability of the findings to more marginalized populations and more diverse pain conditions. This is indicative of the patient population at tertiary pain clinics where participants were recruited from and highlights the importance of recruiting from diverse sites. Future research should replicate these methods in sociodemographically heterogeneous patient populations and in relation to associated pain conditions (e.g., Postural Orthostatic Tachycardia Syndrome/POTS) to further understand patterns of allostatic load expression, adversity exposure, mental and physical health, and repression/social desirability. The current study also lacked comprehensive allostatic load assessment across all relevant areas (e.g., metabolic, inflammatory, autonomic function), which limited our ability to replicate the methodology of the adult study that informed this work. Similar to other contemporary AL studies, sample quartiles were used in place of clinical cutoffs, which may not accurately capture true biological dysfunction. Additionally, risk associated with the upper and lower quartiles may manifest differently, and future AL models should consider accounting for this heterogeneity.

## 5 Conclusion

In conclusion, this study sought to inform our understanding of how AL can be expressed as phenotypes comprising multiple biomarkers, specifically in youth with chronic pain. Biomarkers related to HPA axis functioning and cardiovascular phenotypes were associated with pain-related psychosocial functioning, including pain catastrophizing and childhood adversity. A phenotype-based approach, combined with measurement of composite AL systems, may provide meaningful insights for translational outcome. Phenotypic profiles could support early identification of health risks, improve screening measures in the absence of overt physiological symptoms, or inform clinical interventions targeted at specific biological systems. This may be particularly helpful in high-risk groups, e.g. youth with adversity, and in pediatric populations where long-term health is concerned. The study also raises questions about how to best assign risk for AL biomarkers in a pediatric population experiencing chronic pain and stress, which should be addressed in future research.

## Funding sources

National Center for Complementary and Integrative Health - NCCIH K23AT010643 (SN).

## CRediT authorship contribution statement

**Aahir Mrittika:** Conceptualization, Data curation, Formal analysis, Writing – original draft, Writing – review & editing. **Catherine Ireland:** Conceptualization, Visualization, Writing – original draft, Writing – review & editing. **Carter R. Petty:** Formal analysis, Supervision. **Michelle Bosquet Enlow:** Methodology, Supervision, Validation, Visualization, Writing – original draft, Writing – review & editing. **Sarah Nelson:** Conceptualization, Data curation, Formal analysis, Funding acquisition, Investigation, Methodology, Resources, Supervision, Validation, Visualization, Writing – original draft, Writing – review & editing.

## Declaration of competing interest

The authors declare the following financial interests/personal relationships which may be considered as potential competing interests: Sarah Nelson reports financial support was provided by National Center for Complementary and Integrative Health. If there are other authors, they declare that they have no known competing financial interests or personal relationships that could have appeared to influence the work reported in this paper.

## Data Availability

Data will be made available on request.
